# Effect of a multi-ingredient supplement on intermittent sprint performance, fatigue perception, muscle damage and immunosuppression in recreational athletes

**DOI:** 10.1186/1550-2783-11-S1-P8

**Published:** 2014-12-01

**Authors:** Fernando Naclerio, Eneko Larumbe-Zabala, Robert Cooper, Allgrove Judith, Ahmad Alkhatib, Daniel Baddeley-White, Sergio Garcia, Tobia Zanotto

**Affiliations:** 1 Centre for Sport Science and Human Performance, School of Science, University of Greenwich, United Kingdom; 2 Fundamentals of Motricity and Sports Training Department, School of Sports Sciences, European University of Madrid, Madrid, Spain; 3 Faculty of Science Engineering and Computing, Kingston University, London, United Kingdom; 4 Academy of Sport and Physical Activity, Sheffield Hallam University, Collegiate Crescent Campus, Sheffield, United Kingdom

## Background

It has been suggested that carbohydrate-protein based multi-ingredient supplements may attenuate exercise induced muscle damage (EIMD) and immunosuppression. This study investigates the effects of a commercially available carbohydrate-protein supplement (MTN) enriched with L-glutamine, L-carnitine-L-tartrate compared to carbohydrate alone (CHO) or placebo (PL), on sprint performance, muscle damage, immunosuppression markers and recovery from an intermittent exercise bout.

## Methods

On three occasions, in a counterbalanced order, 16 recreationally trained males volunteered to ingest a multi-ingredient supplement, a carbohydrate supplement or placebo before, during and immediately after a 90min intermittent repeated sprint test (IRS). Measurements included total sprint time and the rate of perceived exertion (RPE) expressed along the IRS. In addition 15m sprint, creatine kinase, myoglobin, interleukine-6, Salivary α amylase; Neutrophil; Lymphocytes and Monocyte were assessed pre, immediately post, 1h and 24h after exercise. Consent to publish the results was obtained from all participants.

## Results

Total sprint times were not different between conditions. RPE increased during the IRS for all conditions, however MTN showed a significant (p<0.001) lower value at the end (15.9±1.4) compared to PL (17.8±1.4) but not with respect to CHO (17.0±1.9). 15m sprint time was reduced (p<0.05) at post, 1hr and 24hr compared to pre with no differences between conditions (p>0.05). Myoglobin increased (p<0.05) in all three conditions at post, and 1hr compared to pre, showing lower values at 1hr (p<0.05) for the CHO and MTN compared to PL (241.8±142.6 ng^.^ml^-1^ and 265.4±187.8 ng^.^ml^-1^ vs. 518.6 ± 255.2 ng^.^ml^-1^ respectively). Interleukin-6 was significantly increased at post and 1h compared to pre (p<0.05) being significantly higher for MTN at post (5.2pg.ml^-1^) and 24hr (2.4pg.ml^-1^) respect to CHO (4.5±2.1 and 1.9±2.5 pg.ml^-1^) but not respect to PL (4.9±2.4 and 1.8±2.4 pg.ml^-1^). Creatine kinase peaks at 24hr for the three conditions with no differences in between them. MTN showed a significant higher Neutrophil concentration (4.9±1.8 10^9^/L) at 1hr compared to CHO (3.9±1.5 10^9^/L) but not to PL (4.5±1.6 10^9^/L).

## Conclusion

Ingesting a multi-ingredient supplement during and immediately after a 90min intermittent repeated sprint test resulted in no effects on performance and higher Neutrophil counts. However, fatigue perception and the accumulation of some muscle damage markers (Mb) could be attenuated.

